# Uncovering Amyloid‑β Interactions: Gray versus White Matter

**DOI:** 10.1021/acschemneuro.4c00439

**Published:** 2025-03-27

**Authors:** Gabriel Cathoud, Mohtadin Hashemi, Yuri Lyubchenko, Pedro Simões

**Affiliations:** † CERES, Department of Chemical Engineering, University of Coimbra, Rua Sílvio Lima-Pólo II, 3030-790 Coimbra, Portugal; ‡ Department of Physics, 12284 Auburn University, Auburn, Alabama 36849-5318, United States; § Department of Pharmaceutical Sciences, University of Nebraska Medical Center, Omaha, Nebraska 68198-6025, United States

**Keywords:** Alzheimer’s disease, amyloid-β, molecular dynamics, membrane–peptide interaction

## Abstract

Alzheimer’s disease is characterized by the accumulation of amyloid plaques in the brain. Recent studies suggest that amyloid-β (Aβ) peptides interact with cell membranes, potentially catalyzing plaque formation. However, the effect of varying cell membrane compositions on this catalytic process requires further investigation. Using molecular dynamics simulations, we demonstrate that a model gray matter membrane significantly influences the secondary structure of β-amyloid peptides. Notably, residues Asp1 and Glu22 play crucial roles in the membrane interaction. Glutamic acid at position 22, located in the middle of the peptide chain, appears to promote the formation of β-hairpin conformations, which are critical for aggregation. Additionally, our simulations reveal that the model white matter membrane allows a spontaneous insertion of segments of the peptide into the membrane, suggesting that membrane interaction not only alters the peptide structure but may also compromise membrane integrity. Our results show that the different membrane compositions in the brain may play different roles when interacting with β-amyloid peptides.

## Graphical abstract

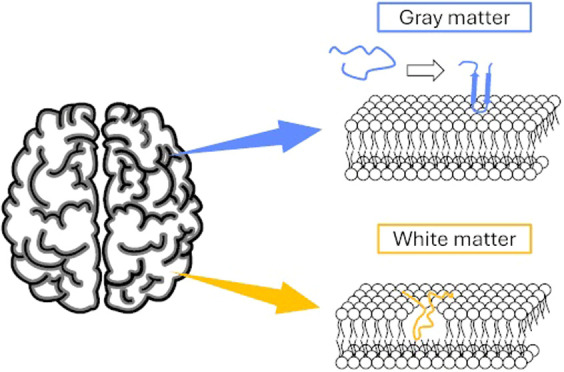

## 1 Introduction

The significant improvements in the quality of life observed in industrialized countries have led to increased life expectancy. While this is a positive development, it has also introduced new public health challenges, particularly the rising incidence of neurodegenerative diseases. Among these diseases, dementia is the most common, affecting a substantial portion of the elderly population. Individuals suffering from dementia face heightened levels of dependency and vulnerability, impacting their physical and mental health as well as their social interactions. It is projected that by 2050, the number of people living with dementia worldwide will reach a staggering 115 million.^1,2^

Alzheimer’s disease (AD) stands out as the leading cause of dementia and globally ranks as the seventh most significant cause of mortality.^3–5^ The primary hallmark of AD is the formation of extracellular amyloid plaques in various regions of the brain.^6^ These plaques originate from the aggregation of amyloid-β (Aβ) peptides, which are produced through the cleavage of amyloid precursor protein (APP).^7–11^ Among the different variants of Aβ peptides, those with 40 and 42 residues (Aβ-40 and Aβ-42) are the most abundant and toxic, respectively.^12–21^

The structural characterization of Aβ peptides is challenging due to their intrinsically disordered nature, which results in a variety of conformations.^22^ Conventional methods struggle to capture these structures accurately, and most insights have been derived from nuclear magnetic resonance (NMR) analysis and molecular dynamics (MD) simulations.^23^ These simulations have proven to be highly valuable in studying the aggregation process, elucidating the various conformations of Aβ peptides, and examining their interactions with different chemical compounds.^23^ Research has indicated that Aβ peptides can form β-strands with flexible regions in between, allowing the formation of β-hairpins.^24^ The formation of these β-hairpins is a critical step in the aggregation process, as it facilitates the formation of β-sheets.^25–27^ These β-sheets can further aggregate, eventually forming amyloid plaques characteristic of AD.

The amyloid cascade hypothesis proposes that the accumulation of Aβ peptides triggers a sequence of events leading to neurodegeneration.^28^ Despite more than a century of research, numerous fundamental questions about AD remain unanswered. For example, the detailed mechanisms underlying the aggregation process of Aβ peptides are still not fully understood.^29^ Addressing these gaps in knowledge is crucial for developing effective treatments and interventions for AD.

Recent studies have highlighted the role of cell membranes in the aggregation of Aβ peptides, suggesting that these membranes might act as catalysts for this process.^29^ Cell membranes are intricate structures comprising two sheets of lipid molecules interspersed with various proteins. Phospholipids are major constituents of membranes and feature both hydrophobic and hydrophilic segments. The hydrophilic groups of the phospholipids may constitute molecules such as choline, serine, or ethanolamine. The phospholipids with serine heads (PS) are usually asymmetrically distributed within the membrane’s leaflets, typically concentrated on the inner side of the lipid bilayer. The PS lipids are exposed on the cell surface in senescent and apoptotic cells. Many neurodegenerative diseases are linked to neuronal apoptosis.^30^

Changes in the lipid composition, due to aging, of healthy human brains (for ages 20–100 years old)^31,32^ show a loss of phospholipids of 42% in females and 43% in males, while cholesterol diminished by 47 and 53%, respectively, when comparing 20 years of age and 100. A more recent study corroborated the findings and further granularized the lipids into specific acyl chain categories.^33^ Additionally, studies have shown that white matter reduction is associated with cognitive impairment.^34–36^ Similar lipid reductions were reported for white matter in the early stages of AD, while disease severity at later stages of disease was linked to the lipid reduction in the gray matter.^37^ Together these findings suggest that the lipid composition changes with age and that disease development coincides with lipid composition changes.

Banerjee et al.^38^ have explored the interaction between Aβ peptides and different lipid membranes using both experimental techniques, such as atomic force microscopy (AFM), and computational methods like molecular dynamics (MD) simulations. Their findings support the hypothesis that lipid membranes can indeed catalyze amyloid peptide aggregation and that the specific composition of these membranes influences the aggregation process. Their research revealed that Aβ proteins could self-assemble into aggregates on phospholipid bilayers under physiological conditions. This process is dynamic, and aggregates are able to dissociate from the membrane, suggesting a complex interplay between Aβ peptides and membrane surfaces that could be critical in the development of neurodegenerative conditions. They also addressed the role of phospholipid composition in the aggregation, showing that aggregation on phosphatidylcholine (PC) bilayer is considerably slower compared to that of a mixed phosphatidylcholine/phosphatidylserine counterpart. Furthermore, they showed that the inclusion of cholesterol in the bilayer dramatically enhances the aggregation propensity of Aβ.^39^ Hashemi et al.^40^ further investigated the role of phospholipid bilayers in Aβ peptide aggregation. Their findings show that the presence of free cholesterol, in addition to the lipid bilayer, accelerates aggregation and enables the Aβ peptide to explore a greater conformation space. The study indicates that particular lipid–Aβ interactions are crucial for the spontaneous formation of neurotoxic aggregates, underscoring the importance of understanding these interactions in detail.

Despite the promising findings from recent experimental and computational studies, the catalytic role of cell membranes in Aβ peptide aggregation has not been thoroughly investigated. In this study, we have explored how different membrane compositions, found in the brain’s white and gray matter, affect the catalytic behavior of cell membranes in the aggregation process. To achieve a more detailed understanding of the Aβ peptide behavior at the atomic level, we employed molecular dynamics (MD) simulations. Our simulations showed an increased affinity of Aβ peptides toward phosphatidylserine (PS)-containing membranes. Special attention must be directed toward the glutamic acid residue at position 22 of the peptide chain, which, throughout all simulations, exhibited prolonged interactions with the membrane. In addition to the interaction with membranes, we also showed that the peptide is inserted into the membrane. This corroborates experimental studies which have shown that the Aβ peptide causes damage to the membrane.^41,42^

## 2 Results and Discussion

We used molecular dynamics simulations to characterize the interaction between Aβ and simple models for white and gray matter, composed of 1-palmitoyl-2-oleoyl-glycero-3-phosphocholine (POPC) and 1-palmitoyl-2-oleoyl-*sn*-glycero-3-phospho-l-serine (POPS), and cholesterol. Three different membrane systems, the composition of each system given in Table S1 (Supporting Information, SI), were used. The simulated membrane systems are named as follows: SYM, for a symmetric lipid composition in the inner and outer leaflet; ASYM, for an asymmetric lipid composition between model; two ASYM systems were used, I, which placed the Aβ peptide facing the inner leaflet of the bilayer, and O, which placed the peptide facing the outer leaflet of the membrane. The Aβ peptide was placed randomly in the simulation box at a center of mass distance of 5 nm to the membrane’s center of mass. Simulations were then carried out for 5 μs; the first 900 ns of all simulations were discarded, and the remaining trajectory was used for analysis.

As expected from its intrinsically disordered nature, Aβ exhibits highly dynamic behavior, frequently changing conformation and moving in a seemingly random manner (see the Movies in the SI). Simulations employed periodic boundary conditions, without any bias or force to prevent crossing of the periodic boundary, which allowed the peptide to freely diffuse from the outer to the inner face of the membrane, or vice versa. Supporting Movies show that the peptide crosses the periodic boundary at approximately 190, 160, and 220 ns for systems gray matter ASYM (O), white matter ASYM (O), and white matter SYM, respectively. For white matter ASYM (I), the peptide undergoes multiple crossings of the periodic boundary before eventually settling for the PS-containing leaflet at 270 ns. For systems gray matter ASYM (I) and gray matter SYM, the peptide does not cross the periodic boundary. Yet, in every simulation, the peptide eventually approached the membrane and remained in contact throughout the remaining simulation time. During these dynamics interactions, the secondary structure for Aβ showed an increased level of formation of β-strands for most of the simulations (Figure 1). Compared to Aβ in water alone, where β-strand formation occurs around the 3 μs mark, in the presence of a membrane, this structural transition starts in less than 0.5 μs in most cases. This significant speed-up in secondary structure transition supports the hypothesis that the membrane acts as a catalyst in the aggregation of Aβ peptides.^29^

**Figure 1:**
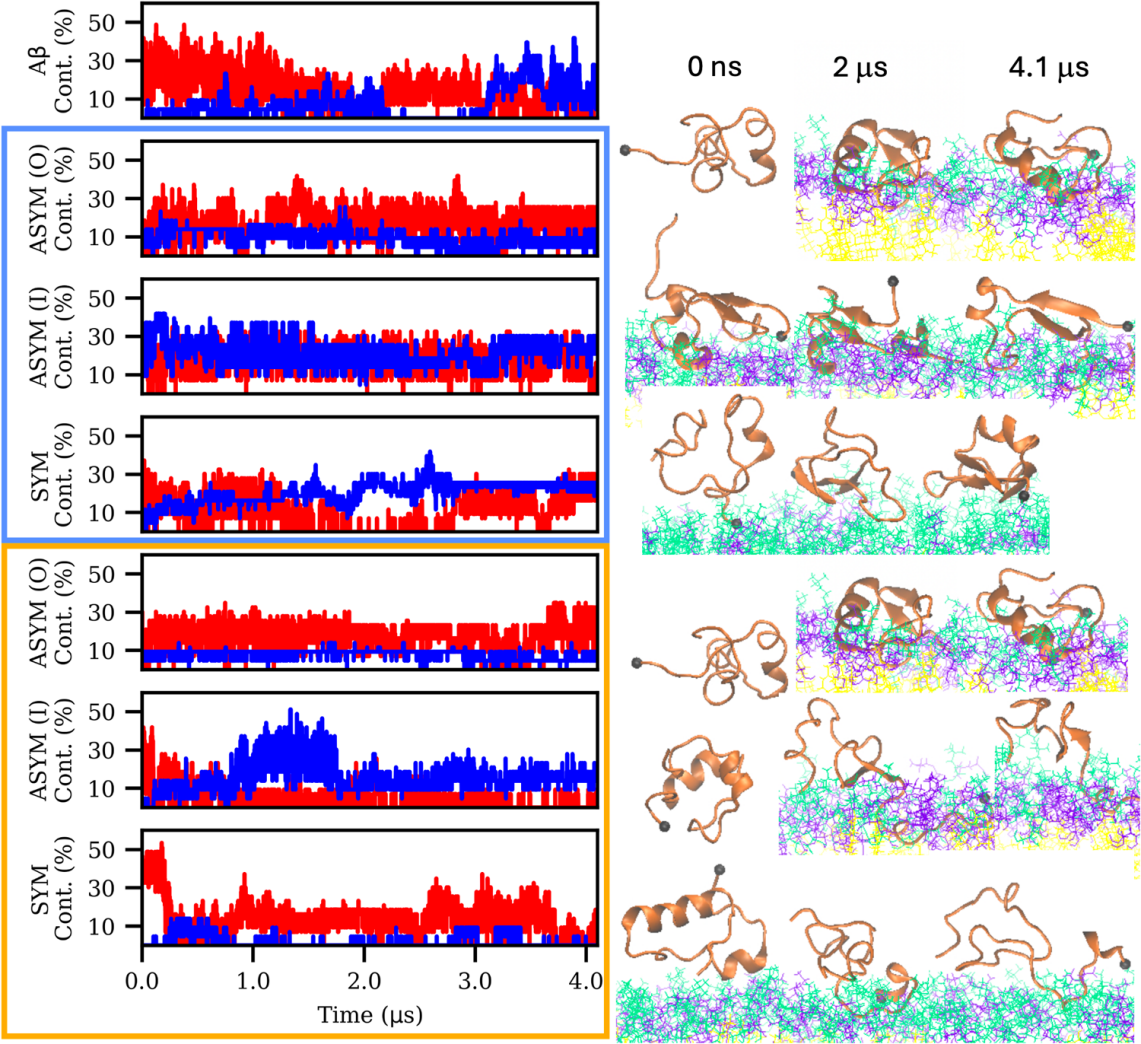
Secondary structure evolution of Aβ in the presence of model white and gray matter lipid membranes. Left, α-helix (red) and β-strand (blue) content over the simulation time. SYM: symmetric membrane model; ASYM: asymmetric membrane model with peptide initially facing inner, I, or outer, O, leaflet; detailed lipid composition is reported in Table S1 (SI). Membranes with gray matter composition are highlighted with a blue box, while an orange box highlights systems with white matter composition. Right, simulation snapshots at 0, 2, and 4.1 μs showing the Aβ peptide in orange, POPC in green, POPS in purple, and cholesterol in yellow, the N-terminal Cα is highlighted with a black sphere. Each row corresponds to the same system.

Comparing the systems with gray and white matter membrane compositions under identical conditions, we found an enhanced formation of β-strands in the gray matter systems. This suggests that the gray matter composition may play a more critical role in the catalytic process of Aβ aggregation. Additionally, we observed the formation of β-strands connected with disordered or helical parts (Figure S1, SI). This conformation is crucial, as it facilitates the formation of β-hairpins, the structure known to lead to peptide aggregation.

Given the intrinsic nature of the peptide, analyzing the distribution of α-helix and β-strand contents allows us to assess the overall tendency of the peptide’s secondary structure (Figure S2, SI). The results show that in many experiments, the membrane and its interaction with the peptide increase the β-strand content over the course of the simulation, which is particularly evident in systems with gray matter composition. Additionally, upon comparison of these distributions with those of the peptide in water alone, it becomes clear that the gray matter composition leads to a higher overall β-strand content.

The analysis of the overall organized content, defined as the sum of α-helix and β-strand contents, further reinforces the conclusion that the membrane presence enhances the structured composition of the peptide (Figure S3, SI). This effect is notably more pronounced in gray matter compositions, suggesting that gray matter plays a more significant role in the conformational change of the peptide and thus in the aggregation process compared to white matter. In the context of lipid reductions due to aging, and specific lipid changes in the brain due to AD, with changes in white matter being more significant in the earlier stages and changes in gray matter being linked with disease severity at later stages,^37^ and based on our findings, which suggest that the interaction of Aβ is different with the model white and gray membranes, we hypothesize that compositional change in brain lipids translates to changed interactions of Aβ with neuronal membranes at different stages of disease development. Furthermore, our findings emphasize how a small change in lipid composition alters the protein–membrane interactions, which ultimately may lead to a change in the aggregation pathway.

To quantify the interaction between Aβ and the membrane, we first assessed the minimum distances between the heavy atoms of the peptide and the heavy atoms of the membrane molecules. The results indicate that the peptide approaches the membrane to distances as close as 3 Å and maintains this proximity throughout most of the simulation time (Figure S4, SI).

Focusing specifically on the α-carbons of the peptide backbone and applying a 6 Å cutoff to account for longe-range interactions, we observed that the segment between residues 20 and 25 shows an increased affinity for membrane molecules in all systems (Figure 2). This finding is crucial, as this central peptide segment may be decisive for the peptide folding in half, leading to the formation of a β-hairpin.

**Figure 2:**
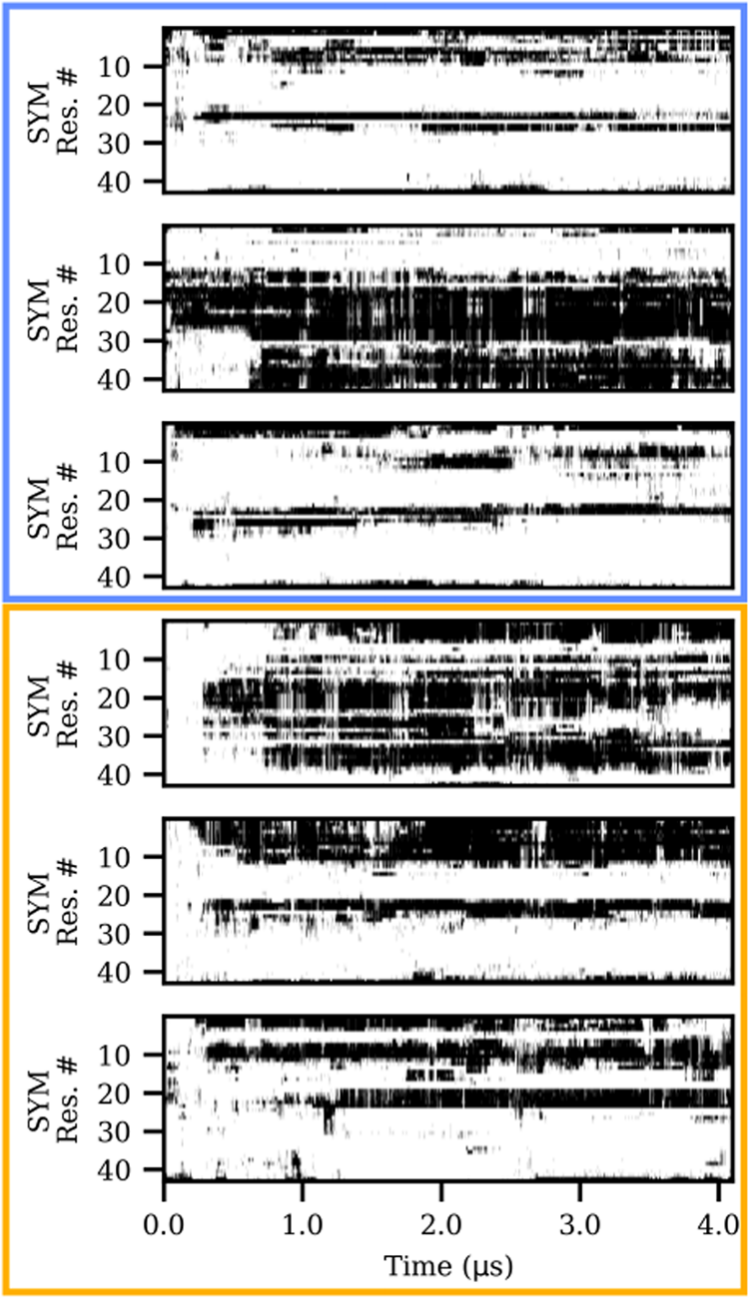
Kymograph of residue-wise contacts with the membrane across the simulations. A cutoff of 6 Å was used between the α-carbons of the peptide backbone and heavy atoms of the membranes. SYM: symmetric membrane model; ASYM: asymmetric membrane model with peptide initially facing inner, I, or outer, O, leaflet; detailed lipid composition is reported in Table S1 (SI). Membranes with gray matter composition are highlighted with a blue box, while an orange box highlights systems with white matter composition.

We then characterized which parts of the membrane the peptide most frequently interacted with. We found that both the phosphate and glycerol groups in the POPC and POPS molecules are the closest to the Aβ peptide (Figure S5, SI). Additionally, the carboxyl and amine groups within PS heads consistently are closer to the peptide, as well. Interestingly, the choline group within the POPC phospholipids exhibits limited proximity to the peptide across all analyzed systems. These findings underscore the peptide’s preference for PS moieties, likely driven by electrostatic interactions.

A residue-wise analysis allows one to discern the interaction preferences for each residue. The average distance to PS head groups in the membranes mimicking the gray matter, shown in Figure 3, reveals that residues 1–6 are typically within 1 nm of the PS heads. For the same membrane, the regions encompassing residues 21–25 are also within 1 nm of the PS heads; furthermore, in the asymmetric configuration starting on the inner leaflet side, the region encompasses residues 19–28. On the membranes mimicking white matter, the N-terminal region exhibits a longer average distance to the PS head groups; however, residues 21–23 are found, on average, within 1 nm of the PS heads. This observation aligns with the findings of Itoh et al.,^43^ who stated that residues 22 and 28 engage with each other due to electrostatic interactions. In light of this, we hypothesize that the interaction with the membrane limits the mobility of the Aβ peptide, thereby making possible long-range interactions, such as Asp1 to Lys28. This can promote the peptide’s folding, thereby facilitating hairpin formation and leading to aggregation. The presence of many different POPS heads across the membrane is likely to be a determining factor in the proximity and positioning of critical residues, potentially leading to enhanced aggregation. Particular attention should be given to the residues in the central region (residues 21–25), which correspond to the peptide segment that is on average closer to the membrane and is crucial for β-hairpin formation. Reducing the freedom of this region of the peptide may allow for the ending parts to come together rapidly. Although this mechanism requires further investigation, it may explain why membranes act as a catalyst in the aggregation process.

**Figure 3:**
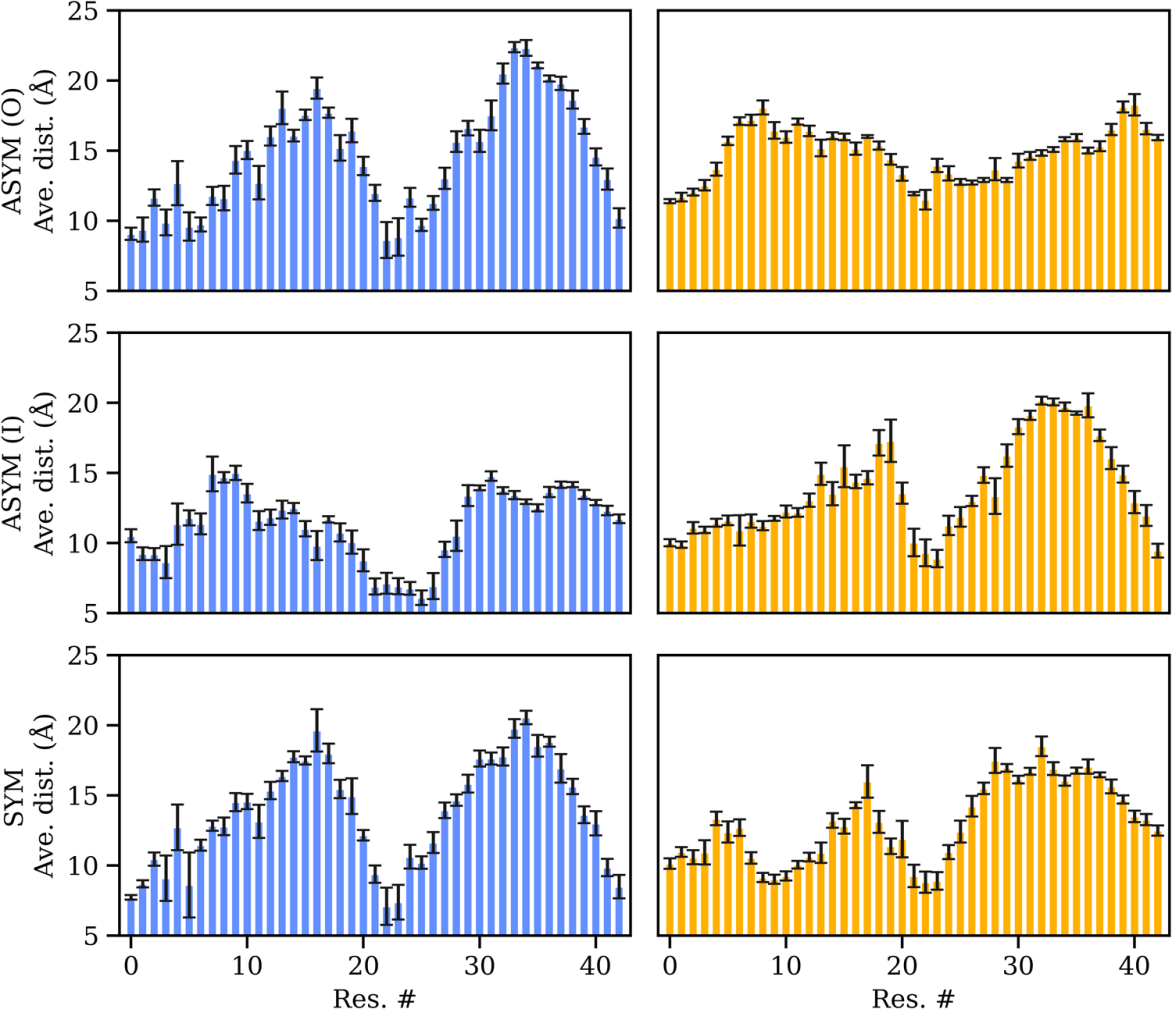
Average distance between each residue in the peptide and the POPS heads of the phospholipids in the membranes. Blue graphs represent systems that mimic gray matter composition, while yellow graphs represent systems mimicking white matter. Error bars represent the standard deviation.

The occurrence and significance of intraneuronal Aβ accumulation in AD have been a topic of considerable scientific debate. While the majority of Aβ is secreted from the cell, it was also localized within various subcellular compartments.^10^ Studies showing that Aβ is initially deposited inside neurons, before being present in the extracellular space, date back to the late 1980s.^44,45^ Our findings, which demonstrate that POPS (phosphatidylserine) significantly enhances membrane interactions of Aβ, and the fact that POPS is predominantly located on the inner membrane of cells, may provide further insight into the early steps of protein aggregation in AD. These results suggest that intracellular Aβ could play a more significant role, potentially being more crucial than extracellular Aβ, in the early stages of the disease.

We then explored the simulations for potential insertion events of Aβ peptides into the membrane. Visual inspection revealed that segments of the peptide were inserted into the membrane in several instances (see Figure 1 and Movies in the SI). Further analysis was performed by calculating the distance between the peptide’s backbone and the plane dividing the membrane by half. By comparing this distance with half of the membrane’s thickness, instances where segments of the peptide were inserting into the membrane are observed (Figure 4). This insertion is particularly pronounced in membranes with a white matter composition. While gray matter composition appears to promote enhanced interaction with the Aβ peptide, white matter facilitates peptide insertion into the membrane. This distinction may be a critical factor in the complex pathogenic pathway of Alzheimer’s disease, in particular because changes in the white matter have been linked to the earlier stages of disease development.^37^ Furthermore, previous studies have indicated that Aβ peptides can induce pore formation in membranes, and experimental studies have documented the insertion of Aβ from bulk solution into the membrane.^41,42^

**Figure 4:**
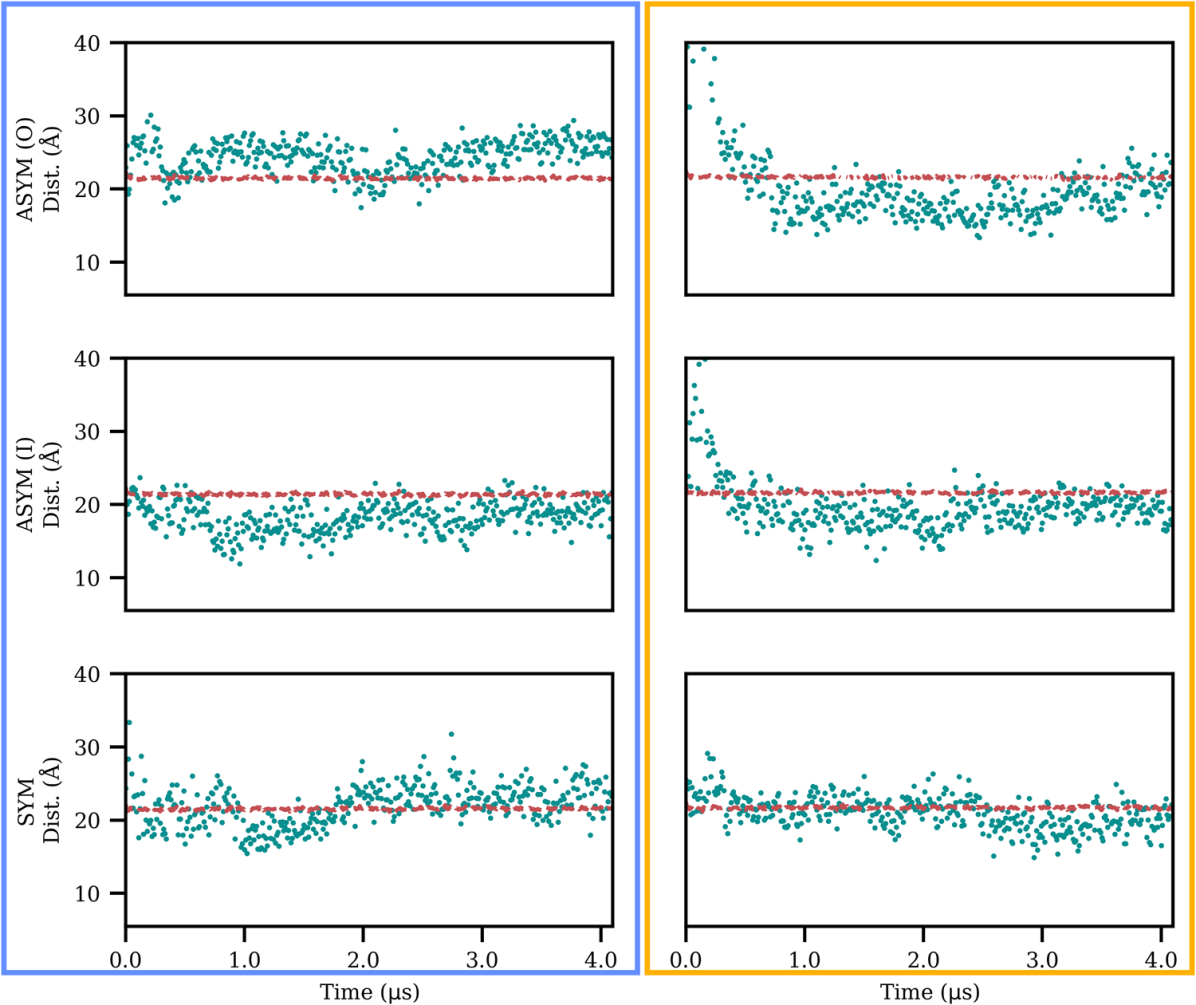
Minimum distance between the peptide backbone and the plane that divides the membrane in half. The dashed red line represents half of the membrane thickness, while the cyan data points represent the minimum distance between any atom in the peptide backbone and the membrane’s dividing plane. SYM: symmetric membrane model; ASYM: asymmetric membrane model with peptide initially facing inner, I, or outer, O, leaflet; detailed lipid composition is reported in Table S1 (SI). Membranes with gray matter composition are highlighted with a blue box, while an orange box highlights systems with white matter composition.

Beyond the behavior of the Aβ peptide and its interactions with the membrane, we also evaluated the free energy landscape using the dihedral space of the peptide’s backbone. To manage the high dimensionality of the torsion angles’ domain, we applied principal component analysis (Figure 5). Using a clustering technique, we extracted conformation clusters based on the data’s density across the two principal components. From the data corresponding to each cluster, an average conformation was derived. In most systems, this average conformation corresponds to the formation of a β-hairpin. Snapshots of the conformation closest to the average, based on root-mean-square deviation, for the lowest-energy clusters are shown in Figure 5 and demonstrate that apart from one system, the major secondary structure elements are β-strands. This finding emphasizes that the presence of the membrane may significantly contribute to the aggregation of the Aβ peptide, reinforcing the idea that membrane interactions are pivotal in the development of neurodegenerative diseases like Alzheimer’s.

**Figure 5:**
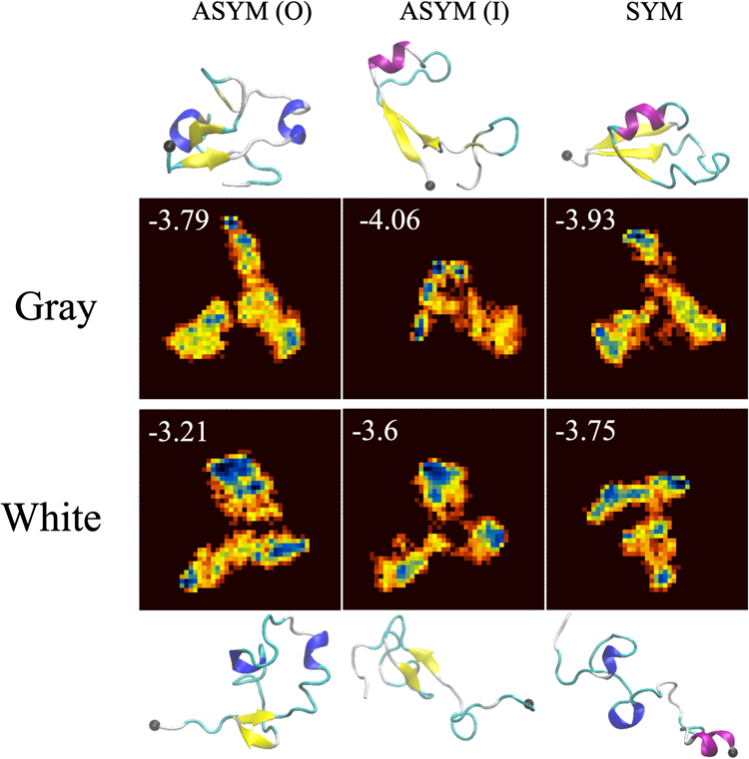
Free energy landscape based on the dihedral principle component analysis. Each plot are free energy of a simulation system based on the first and second dihedral principle components. The lowest energy for each plot is noted in units of kcal/mol. Snapshots are representative conformations for the lowest-energy clusters, with β-strands in yellow, α-helices in magenta, and 3/10-helices in blue.

## 3 Conclusions

Our simulations confirm the hypothesis that the presence of a membrane significantly influences the behavior of amyloid-β peptides, accelerating the formation of β-strands and increasing their organized content. The results highlight an increased affinity of amyloid-β peptides toward phosphatidylserine heads of the phospholipids present in the membranes, likely driven by electrostatic interactions due to the charged nature of these lipids. Special attention must be directed toward the glutamic acid residue at position 22 of the peptide chain. Its central location within the peptide suggests it may act as a pivotal point, facilitating the peptide’s folding in the middle and promoting the formation of hairpin structures. In addition to these interaction features, this study uncovered an intriguing phenomenon where the peptide is inserted into the surface of the membrane. This finding offers new insights into the aggregation dynamics of Aβ peptides and sheds light on the complex pathways underlying Alzheimer’s disease. Notably, the gray matter composition exhibits a more pronounced effect on peptide behavior compared to white matter composition, indicating distinct roles for various brain regions in Alzheimer’s Disease.

## 4 Methods

We elected to use simple models with POPC, POPS, and cholesterol (CHOL) to elucidate which abundant lipid is most important for the membrane interaction of Aβ, whose composition was based on the reported values for a 55-year-old human brain.^46,47^ This specific age was selected due to the correlation between the occurrence of AD and individuals’ age. Four membrane models were prepared: two symmetrical, i.e., with the same composition in both leaflets, and two asymmetrical. A total of 512 phospholipids (POPC and POPS) and 102 CHOL molecules were used for each membrane. Since POPS heads remain at the inner side of a membrane during the normal functioning of a cell,^30^ the asymmetric membrane models were prepared including one leaflet composed of all POPS molecules and the other with only POPC molecules. The latter were distributed so that the number of phospholipids in each leaflet was the same. The composition of each system is detailed in Table S1 (SI).

The membrane models were prepared using the CHARMM-GUI tool (www.charmm-gui.org).^48–50^ CHARMM-GUI distributes the peptides in a bilayer, considering the expected area per lipid for the different lipid molecules. The force field lipid21 from the Amber force field package was used for lipids.^51^ The simulation boxes were prepared with the membrane coordinate file obtained after a preparatory step. The *X* and *Y* dimensions of the box were kept the same as the original membrane coordinate file (values around 12 by 12 nm), while the *Z* dimension was set to 16 nm for all of the systems for uniformity.

The Aβ-42 peptide initial conformation was obtained from previous studies by Hashemi et al.,^40^ while its protonation state was set to match pH 7. An additional cysteine was added at the beginning of the chain (position 0), resulting in a chain with 43 residues. This was used to mimic experimental conditions where CYS was used to attach the peptide to fluorophores or substrates for AFM or fluorescence studies. Controls have shown that the additional CYS does not affect the Aβ behavior or its aggregation process.^52^

Recent developments of amyloid-specific improvements for force fields have elucidated the effects of force field choice on the simulation results for amyloid proteins.^53–55^ These studies have made changes in the protein–protein or protein–solvent interaction potentials and achieved an improved correlation between simulation and experimental results. However, further validation is required of the force field modification as no study has characterized the effects of the modifications on the combination of force fields, e.g., protein and lipid force fields. For this reason, we selected the AMBER99SB-ILDN force field to model the topology of the Aβ.^56^ The peptide was inserted randomly in the simulation box but with a 5 nm distance between its center of mass and the membrane’s center of mass. The obtained box was then solvated using GROMACS,^57^ and the water molecules inside the membrane were removed using the VMD software (www.ks.uiuc.edu/Research/vmd/).^58^ Cl^–^ and Na^+^ ions were added to neutralize the system, given the negative charge of the POPS molecules and to add an ionic concentration of 0.15 M to the bulk.

All of the simulations comprised minimization, simulated annealing, NVT and NPT equilibration, and NPT production steps. A temperature of 300 K was used to reproduce experimental conditions for Aβ-supported bilayer experiments. The simulated annealing gradually heated the system to the desired temperature by using seven annealing points, each consisting of 20 ps. In the last step, i.e., at 300 K, the annealing time was 100 ps. The bath temperature was increased by 50 K for each annealing point, starting with 0 K up to 300 K. The V-rescale^59^ thermostat was used. The NVT equilibration consisted of a 500 ps simulation, and, again, the V-rescale^59^ thermostat was used. The NPT equilibration consisted of a 10 ns simulation, and the C-rescale^59^ barostat was used for a semi-isotropic pressure coupling. The temperature coupling was done using again the V-rescale^59^ thermostat.

In the production step, the Parrinello–Rahman barostat^60^ and the V-rescale^59^ thermostat were used for pressure and temperature coupling, respectively. The systems were simulated for 5 μs. The first 900 ns of all simulations were discarded, and the remaining trajectory was used for analysis. All minimization and equilibration steps were performed with GROMACS software.^57^ The production step was carried out for 900 ns with GROMACS and the remaining part on an Anton 2 supercomputer.

The secondary structures of the peptide were determined using the DSSP tool implemented in the MDTraj package.^61^ This analysis used a simplified notation, categorizing the residues as constituents of either α-helix, a β-strand, or a coil, the latter representing an unstructured conformation. Based on the data derived from the DSSP tool, the cumulative content of both the α-helix and β-strand was calculated by counting the residues associated with each secondary structure type and dividing the result by the total number of residues in the peptide chain to obtain a normalized result.

The distances between atoms were calculated using the MDAnalysis package.^62,63^ The peptide–membrane distance was defined as the shortest distance between the atoms of the peptide’s backbone and any heavy atom within the membrane. The insertion occurrence was assessed by measuring the shortest distance between the peptide and the plane that divides the membrane in half and comparing this distance with half of the membrane’s thickness. The plane was defined by the *X*–*Y* plane, which contains the center of geometry of the membrane. The center of geometry was computed from the selections of the phospholipid heads, encompassing choline, serine, phosphate, and glycerol groups. The membrane’s thickness was calculated using the LiPyphilic^64^ tool.

The free energy landscape was analyzed by using the Carma package. In this case, the ϕ and ψ angles were considered. Furthermore, based on the results for the principal components and distribution of the conformations data across the principal components, Carma identifies clusters centered around densely populated regions. With conformations of each cluster, it also creates a .pdb file containing the average positions of the residues in each cluster.

## Supplementary Material















## Notes

Collection of supplementary results derived from the study, highlighting significant findings and observations that complement the primary outcomes (PDF)
Peptide-membrane interaction in System 1 (MOV)
Peptide-membrane interaction in System 2 (MOV)
Peptide-membrane interaction in System 3 (MOV)
Peptide-membrane interaction in System 4 (MOV)
Peptide-membrane interaction in System 5 (MOV)
Peptide-membrane interaction in System 6 (MOV)
