# Supplementary material for: Uncovering Amyloid‑β Interactions: Gray versus White Matter
Source: ACS Chem Neurosci. 2025 Mar 27;16(8):1433–41. doi: 10.1021/acschemneuro.4c00439 (PMC12818754; doi:10.1021/acschemneuro.4c00439)
Supplement: Supplementary file 1 [file cn4c00439_si_001.pdf]

Uncovering amyloid-beta interactions: gray *versus* white matter

Supporting Information

Gabriel Cathoud,[†] Mohtadin Hashemi,[*,‡] Yuri Lyubchenko,[¶] and Pedro Simões[*,†]

†*University of Coimbra, CERES, Department of Chemical Engineering, Coimbra, Portugal*

‡*Department of Physics, Auburn University, United States of America*

¶*Department of Pharmaceutical Sciences, University of Nebraska Medical Center, United States of America*

E-mail: hashemi@auburn.edu; pnsim@eq.uc.pt

Videos

The simulation videos are available for download from **this link**.

Figures & Tables

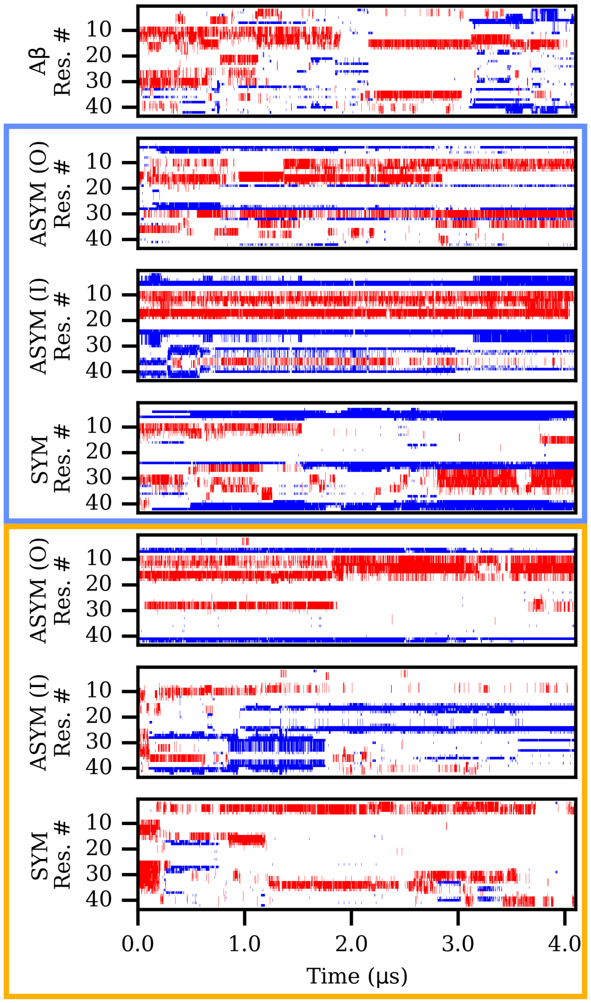

Figure S1: (α-helix (red) and β-strand (blue) parts in the peptide chain over the simulation.

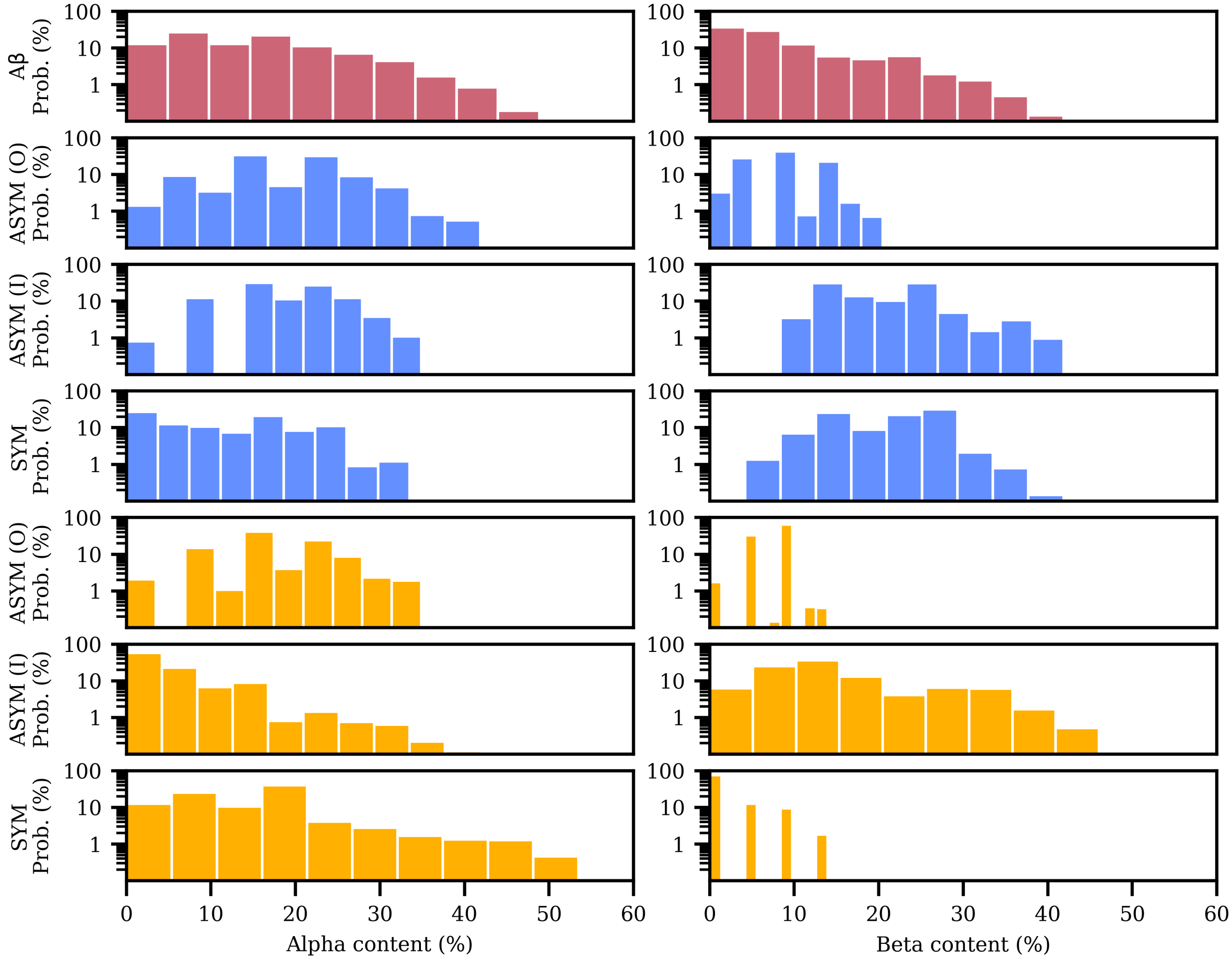

Figure S2: Distribution of the α-helix (red) and β-strand contents over the simulation.

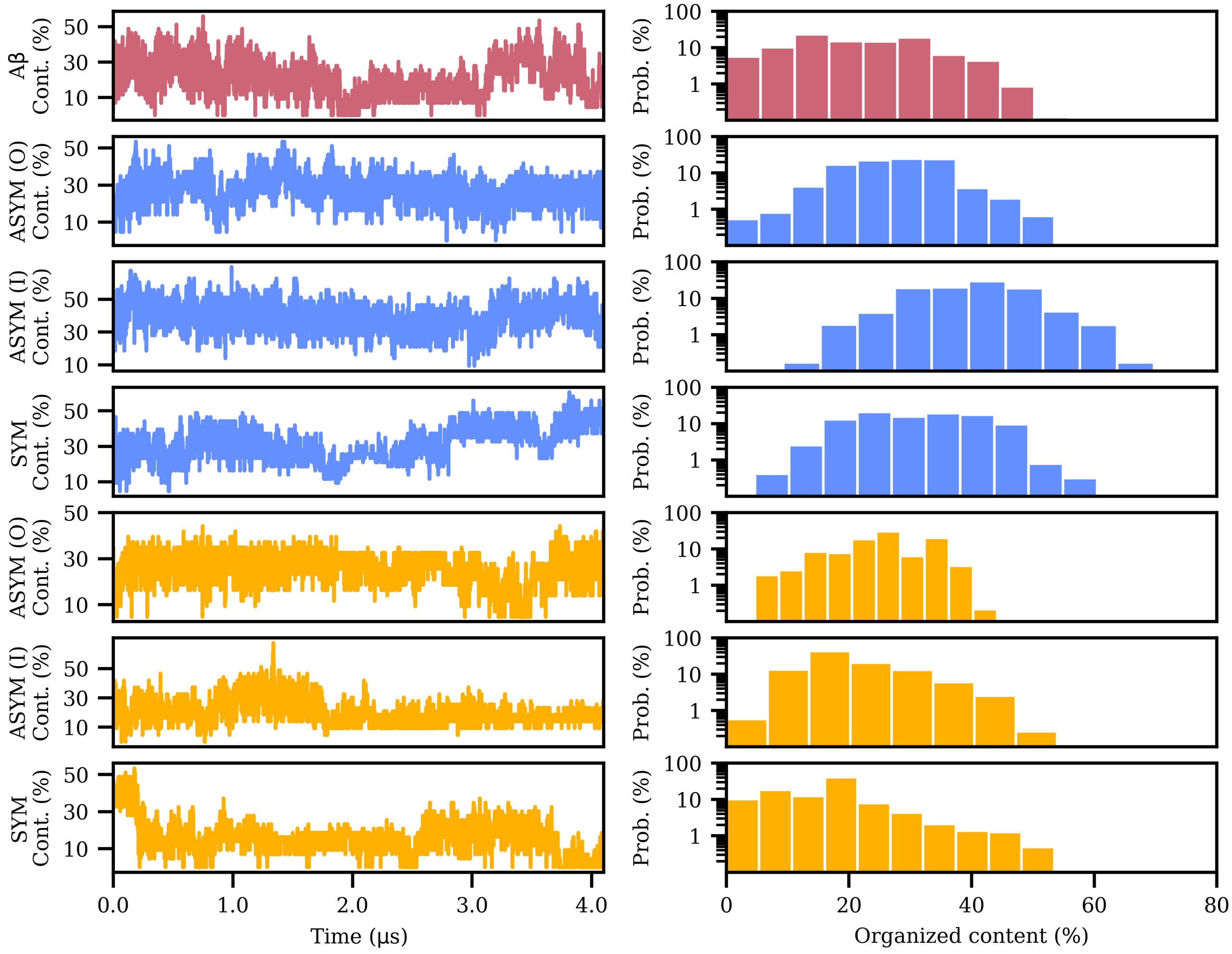

Figure S3: Organized content in the peptide's chain across the simulation (left) and its distribution over the simulation (right).

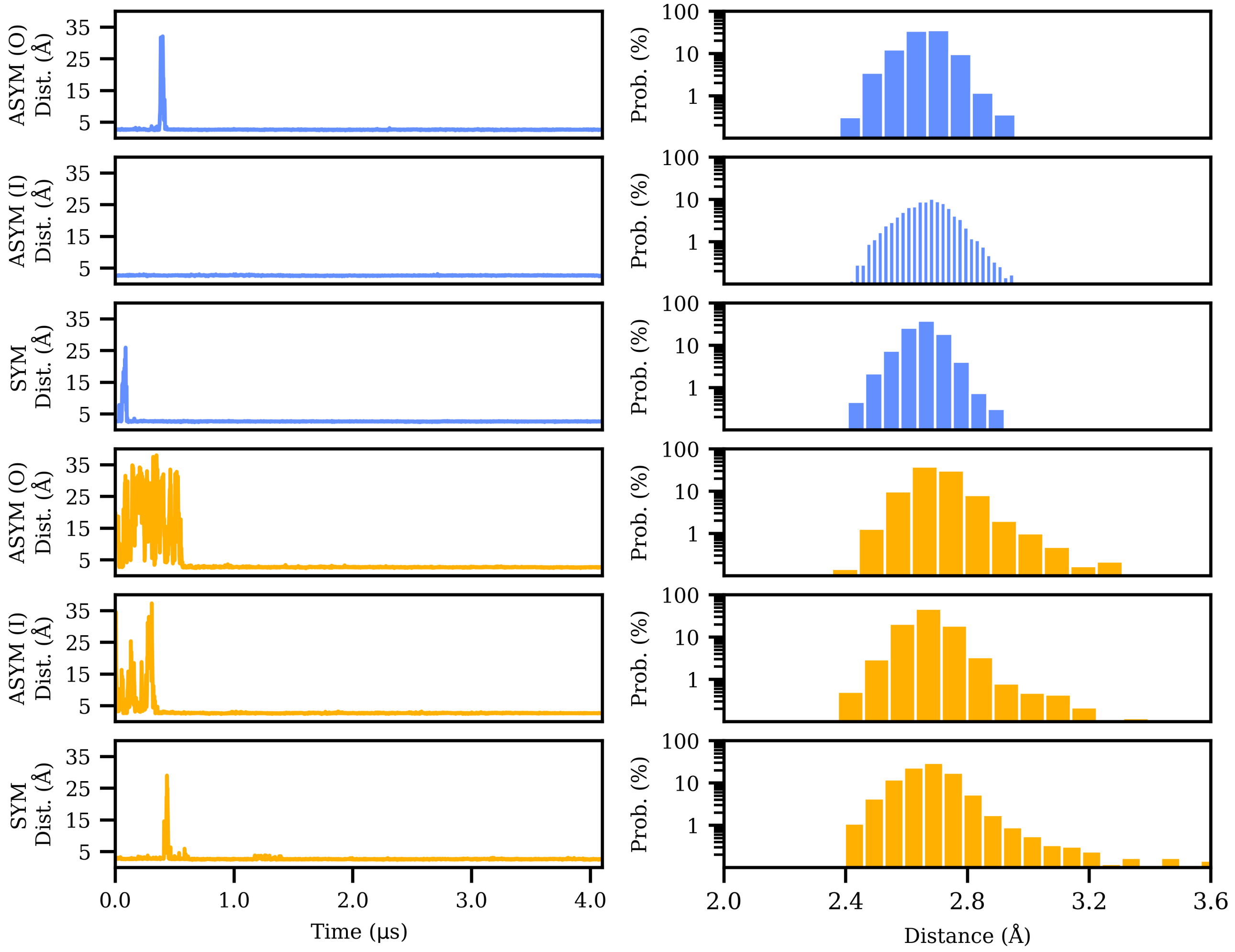

Figure S4: Shortest distance between the peptide and the membrane (left) and its distribution over the simulation (right).

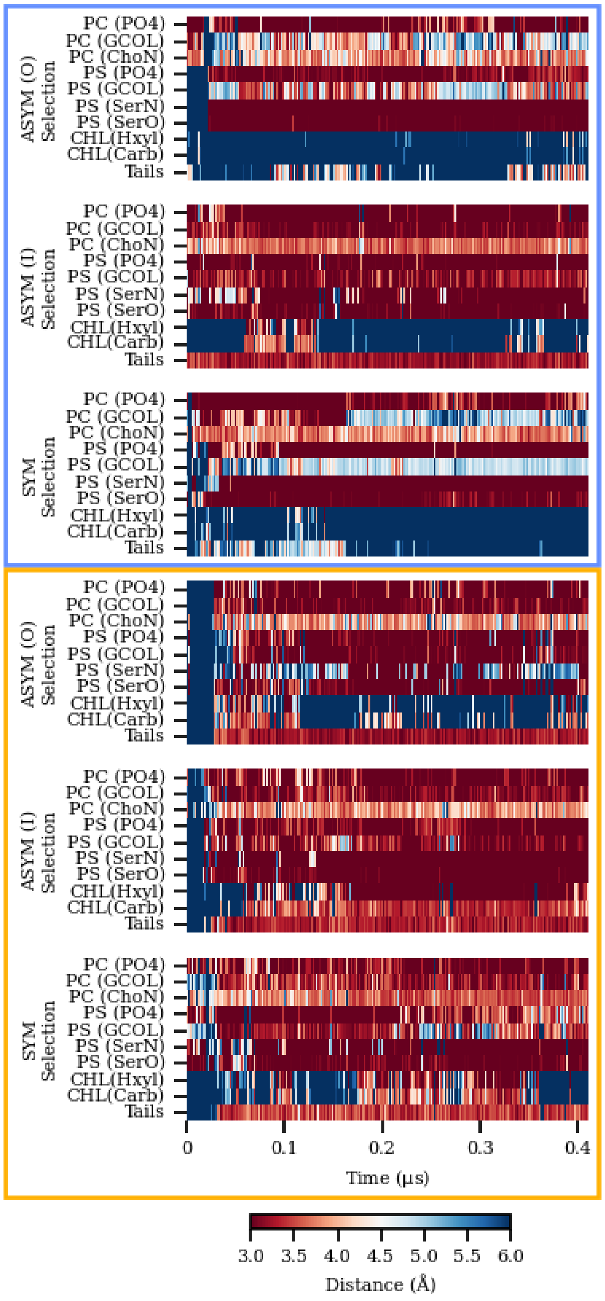

Figure S5: Shortest distance between the peptide and different selections of the membrane across the simulation.

Table S1: Atomic compositions used in the simulations.

System GR ASY (O)			System WH ASY (O)		
	Number of molecules	Number of atoms		Number of molecules	Number of atoms
POPC	396	53064	POPC	340	45560
POPS	116	14732	POPS	172	21844
CHL	102	7548	CHL	102	7548
Na^+	-	343	Na^+	-	394
Cl^-	-	224	Cl^-	-	219
Water	56569	169707	Water	55433	166299
Aβ	1	638	Aβ	1	638
Total	57184	246256	Total	56048	242502
Box dimensions:	124.44x124.44x160.00 Å		Box dimensions:	123.22x123.22x160.00 Å	
Box volume:	2477.69x10^3 $Å^3$		Box volume:	2429.11x10^3 $Å^3$	

System GR ASY (I)			System WH ASY (I)		
	Number of molecules	Number of atoms		Number of molecules	Number of atoms
POPC	396	53064	POPC	340	45560
POPS	116	14732	POPS	172	21844
CHL	102	7548	CHL	102	7548
Na^+	-	343	Na^+	-	394
Cl^-	-	224	Cl^-	-	219
Water	56663	169989	Water	55383	166149
Aβ	1	638	Aβ	1	638
Total	57278	246538	Total	55998	242352
Box dimensions:	124.44x124.44x160.00 Å		Box dimensions:	123.22x123.22x160.00 Å	
Box volume:	2477.69x10^3 $Å^3$		Box volume:	2429.11x$10^3$$Å^3$	

System GR SYM			System WH SYM		
	Number of molecules	Number of atoms	Number of molecules	Number of atoms	
POPC	396	53064	POPC	340	45560
POPS	116	14732	POPS	172	21844
CHL	102	7548	CHL	102	7548
Na^+	-	344	Na^+	-	394
Cl^-	-	225	Cl^-	-	219
Water	56577	169731	Water	55216	165648
Aβ	1	638	Aβ	1	638
Total	57192	246282	Total	55831	241851
Box dimensions:	124.70x124.70x160.00 Å		Box dimensions:	123.07x123.07x160.00 Å	
Box volume:	2488.09x$10^3$$Å^3$		Box volume:	2423.36x$10^3$$Å^3$	
